# Impact of Pandemic-Related Social Restrictions on Language and Speech Development in Children with Cleft Lip and/or Palate at 18-24 Months

**DOI:** 10.1177/10556656251328843

**Published:** 2025-03-28

**Authors:** Amy Davies, Lucy Southby, Sharon Baker, Helen Extence, Neil Brierley, Yvonne Wren

**Affiliations:** 1 The Cleft Collective, Bristol Dental School, 1980 University of Bristol, Bristol, UK; 2 Bristol Dental School, 1980 University of Bristol, Bristol, UK; 3 Bristol Speech and Language Therapy Research Unit, 159003 North Bristol NHS Trust, Bristol, UK; 4 The Welsh Centre for Cleft Lip and Palate, 7757 Swansea Bay University Health Board, Port Talbot, UK; 5 West Midlands Cleft Service, 1729 Birmingham Children's Hospital, Birmingham Women's and Children's NHS Foundation Trust, Birmingham, UK; 6 Cardiff School of Sport and Health Sciences, 11352 Cardiff Metropolitan University, Cardiff, UK

**Keywords:** cleft lip, cleft lip and palate, cleft palate, speech development, language development

## Abstract

**Objective:**

Determine if early speech and language development in children born with cleft lip and/or palate (CL/P) was impacted by social restrictions during the COVID-19 pandemic.

**Design:**

Cohort study using data from The Cleft Collective.

**Participants:**

Children with CL/P whose first 18-24 months were before the pandemic compared to children whose first 18-24 months were impacted by the pandemic.

**Measures:**

Primary outcome: parental reported Ages and Stages Questionnaire—Third Edition (ASQ-3). Secondary outcomes: 18- to 24-month speech and language therapy (SLT) assessment location; ability to judge velopharyngeal function for speech; SLT judged-expressive language and consonant inventory size for children with a cleft palate. Analyses adjusted for confounders.

**Results:**

We found no statistical evidence to suggest a difference in ASQ-3 communication (n = 631; OR_adjusted_ 0.96; 95% CIs 0.533, 1.742; *P* = 0.902), SLT judgements of expressive language (n = 175; OR_adjusted_ 0.66; 95% CIs 0.224, 1.947; *P* = 0.452), or consonant inventory size at age 18-24 months (n = 186; IRR_adjusted_ 0.98; 95% CIs 0.775, 1.249; *P* = 0.896) between pre-pandemic and pandemic impacted groups. There was weak statistical evidence of an association between virtual appointments and SLT being unable to rate features of velopharyngeal function for speech (OR_adjusted_ 3.54; 95%CIs 0.849, 14.755; *P* = 0.083).

**Conclusions:**

No statistical evidence of an association between exposure to pandemic-related social restrictions and early language development or consonant inventory size at age 18-24 months. Small sample sizes, variation in the pandemic impacted groups, and limitations of the measures should be considered when interpreting the findings. Further work is required to further examine the use of virtual appointments for speech assessment.

## Introduction

Children's communication environment is important for their early speech and language development.^1^ There is evidence that attendance at early care and education settings is beneficial for speech, language, and communication (SLC) development for children in all socioeconomic groups,^2,3^ with opportunities to interact with peers in the early years potentially enhancing language development through negotiation, planning, and joint interactions.^4^ Parents’ interactions with their child are also a key influencing factor for language learning in the early years,^5,6^ and there is some evidence that maternal depression and anxiety have a negative association with children's language development.^7^

### Impact of the Pandemic on Children's Communication Environment

The COVID-19 pandemic brought changes to children's everyday routines and interactions due to lockdowns and social distancing measures, potentially changing their access to, and experiences of, the stimulation and input they would usually receive for their early SLC development.^8^ Opportunities to interact with their peers were limited due to closure of formal early care and education settings, and in less formal settings through closures and social distancing measures e.g., parent-toddler groups, parks, and play centers. Children's interactions with their wider family networks may also have been reduced, and parents reported an increased level of stress and anxiety,^9,10^ with concerns reported regarding finances; closure of schools; and health needs of themselves and their friends and family.^11^ These factors could have had a negative impact on children's SLC development.

### Impact of Social Restrictions on Speech, Language and Communication Development

Pandemic-related social restrictions may have facilitated opportunities for early language development in some cases where parents were spending more time at home.^12^ However, there have been reports that the SLC skills of children impacted by the pandemic restrictions at later pre-school age and just prior to school entry were negatively affected.^13–15^ Evidence regarding impacts on earlier SLC development in children up to age 24 months is variable.

Studies of 12-month-old children in the general population impacted by the pandemic have reported some evidence that fewer were using one meaningful word and pointing and waving goodbye.^16^ Moreover, scores on the Ages and Stages Questionnaire—Third edition (ASQ-3)^17^ in a meta-analysis of available studies^18^ suggested that children were at higher risk of communication difficulties when compared to pre-pandemic cohorts. A study^19^ examining ASQ-3 communication domain categorization in large groups of children at ages 10 and 18 months, compared children from before the pandemic with three groups with different amounts of exposure to the pandemic and associated restrictions. The first “pandemic” group were 12 months old at the start of the pandemic so had no exposure at age 10 months but some exposure by 18 months; in the second “pandemic” group some but not all would have been exposed by age 10 months and all by age 18 months, and all children in the third “pandemic” group were born at or after the start of the pandemic so were exposed at both age 10 months and 18 months.^19^ They found evidence for increased odds of being categorized as requiring onward referral in the second and third pandemic groups at age 18 months.^19^ At age 10 months, there was evidence of increased odds of being in the referral category on the communication domain in the third pandemic group when analyses were adjusted for demographics and family risks.^19^ In contrast, a study^20^ found that while ASQ-3 communication domain categorization differed between pre- and post-pandemic groups at age 6 and 12 months, there was no statistical evidence to suggest a difference between groups at age 18 or 24 months. A further study^12^ did not find evidence for an association between increased exposure to the pandemic (number of days between pandemic onset and child turning age 12 months) and scores on the Language ENviroment Analysis (LENA) Developmental Snapshot^21^ in longitudinal data for a group of children at ages 12 and 24 months.

The variability in the findings described above may be influenced by variation in exposure to pandemic-related restrictions between pandemic impacted study populations, variation between pre-pandemic comparison cohorts, and the range and nature of measures used to examine early language and communication development across studies. There may also be variation between studies due to sample sizes and participant demographics.

### Potential Impact of Social Restrictions on Speech, Language, and Communication Development in Children Born with a Cleft

Children born with cleft palate with or without cleft lip (CP+/-L) are at increased risk of their language development being delayed in comparison to their peers as well as disordered speech.^22^ They are also at increased risk of glue ear and conductive hearing loss^23,24^ which can impact upon SLC development.^25,26^

The potential risks to early SLC development presented by pandemic-related social restrictions described above also applied to children born with CP+/-L. Restrictions also meant that much of the support available via health services for children born with CP+/-L, including speech and language therapy (SLT), was delayed or provided virtually through telehealth services^27,28^ resulting in some anxiety and frustration for parents.^29^ Routine assessment and monitoring of hearing for children with CP+/-L could not be provided through telehealth services and so it was not possible to assess or provide intervention for hearing difficulties.^27^ It is therefore possible that the pandemic restrictions added risks to the SLC development of children born with CP+/-L, in addition to those already present for this population.

Routine pathways are already in place in the United Kingdom (UK) and elsewhere to monitor speech and language development in children born with CP+/-L. However, understanding whether young children born with cleft lip and/or cleft palate (CL/P) who were impacted by the pandemic were more likely to experience difficulties in their early SLC skills compared to children with CL/P who were not impacted by the pandemic will help inform services and other stakeholders as these children approach full-time school entry. It also adds to the evidence regarding the impact of social restrictions due to a global pandemic.

The aim of this study was therefore to examine whether early speech and language development in children with CL/P age 18-24 months who were impacted by social restrictions during the pandemic, was different to that of children with CL/P who were age 18-24 months before the start of the pandemic.

## Method

Existing data from a longitudinal prospective birth cohort study, The Cleft Collective, were analyzed to address the aim of this study (Cleft Collective project number CC050-LS). The Cleft Collective is a large prospective cohort study of children born with cleft lip and/or palate (CL/P), investigating causes of cleft, the best treatments and the impact of cleft on those affected and their families. The resource comprises biological samples, speech audio recordings, medical and educational records, and parent and child completed questionnaires. The resource is available for clinical and academic communities to access and use to address a range of cleft-related research questions.^30^ More information on the study and how to access the dataset is available at http://www.bristol.ac.uk/cleft-collective/professionals/access/. Funding for the Cleft Collective was provided by The Scar Free Foundation, Vocational Training Charitable Trust, and The Underwood Trust.

Ethical approval for the analyses reported in this paper was granted by the Faculty of Health Sciences Research Ethics Committee at the University of Bristol; Reference 13848. Ethical approval for the Cleft Collective was obtained from the South West National Research Ethics Service (REC 13/SW/0064).

### Cleft Collective Participants

Participants in the Cleft Collective cohort study are recruited from all regional specialist cleft centers in the UK. For the birth cohort, parents of babies born with CL/P are recruited either before or soon after birth; children are recruited before their first primary surgery. For the 5-year cohort, parents and children born with CL/P are recruited within the year after the child's fifth birthday.^30^ Data from the Cleft Collective birth cohort were utilized within this study. The data analyzed for this study came from parent questionnaires sent to participating families of children born with CL/P when their child turned 18 months of age, and from routine SLT assessments carried out when children of participating families, born with CP+/-L, were aged between 18-24 months.

### Inclusion Criteria for the “Pre-Pandemic” and “Pandemic Impacted” Groups

Cleft Collective participants were included in the “pre-pandemic” group if their 18-month parent questionnaire and/or the 18- to 24-month SLT assessment were completed before the start of the first national lockdown in the UK on March 23, 2020.^31^ The “pandemic impacted” group included child participants in the Cleft Collective who were born no more than 6 months before the first national lockdown and whose 18-month parent questionnaire and/or 18- to 24-month SLT assessment were completed before July 1, 2022, 4 months after the end of all legal restrictions in England. Data for the pre-pandemic group were collected over a period of 6 years and data for those within the pandemic impacted group were collected over a period of 16 months.

### Parent Questionnaire Data

For the primary analysis, data for the communication domain of the ASQ-3^17^ were taken from the mother's questionnaires sent to families of children with CL/P participating in The Cleft Collective when their child turned 18 months of age. The ASQ-3 is a screening questionnaire of children's development. Data for the communication domain only were used for this study. The communication domain comprises six questions with specific examples asking whether the child understands or uses a particular language concept or communication skill. Response options are either “yes,” “sometimes” or “not yet.” Numerical scores allocated to each rating are combined to generate a score for the domain. Domain scores were subsequently categorized as either “on schedule,” “requires monitoring” or “requires onward referral” as per ASQ-3 guidance.^17^

### Speech and Language Therapy Assessment Data

In the UK, children born with CP+/-L are routinely seen for SLT assessment between the ages of 18-24 months. Data from these assessments for children participating in The Cleft Collective Speech and Language study (a subsample of the main Cleft Collective birth cohort)^32^ were analyzed. Specifically examined were SLTs judgements of, consonant inventory size (number of different consonant sounds heard at assessment), receptive and expressive language development (assessed informally through play and observation), presence of active cleft speech characteristics and velopharyngeal function for speech including resonance, airflow and presence of passive cleft speech characteristics.

### Confounding Variables

Variables included as potential confounders in adjusted analyses were cleft subtype, syndromic status inclusive of children with non-syndromic Pierre Robin Sequence (PRS), biological sex, socioeconomic status (SES), maternal report of diagnosed hearing loss or impairment, the child's age at completion of the 18-month questionnaire, and the child's age at the time of primary palatal surgery. SES was derived from the Indices of Multiple Deprivation (IMD).^33^ The IMD uses information on employment, education, health crime, barriers to housing and services, and living environment associated with area postcodes (UK equivalent to a Zip Code) to categorize postcodes into one of 10 deciles, one being the most deprived decile and 10 being the least deprived.

### Primary Analyses

Descriptive statistics were undertaken to explore the distribution of the ASQ-3 communication domain at 18 months of age and the frequency and percentage of children classified as pre-pandemic and pandemic impacted and other sample characteristics. Sample characteristics explored included cleft type (cleft lip only / cleft palate with or without cleft lip), syndromic status excluding non-syndromic PRS, syndromic status inclusive of children with non-syndromic PRS, biological sex, SES, hearing status, child's age at completion of the 18-month questionnaire, age at completion of the 18- to 24-month SLT assessment, and the child's age at the time of primary palatal surgery. Categorical sample characteristics were compared between pre-pandemic and pandemic impacted children using chi-square and ordinal chi-square tests where appropriate. Mann-Whitney U tests were undertaken to identify if differences occurred in the distribution of child's age at completion of the questionnaire and child's age at primary palatal repair between the pre-pandemic and pandemic impacted categories.

The distribution of ASQ-3 communication domain was compared between pre-pandemic and pandemic impacted children and categorical sample characteristics using ordinal chi-square tests. Kruskal-Wallis tests were undertaken to identify if differences occurred in the distribution of child's age at completion of the questionnaire and child's age at primary palatal repair between the ASQ-3 communication domain categories. Further exploration into the association of ASQ-3 communication domain between pre-pandemic and pandemic impacted children was undertaken using ordinal logistic regression. One unadjusted (model 1) and two adjusted ordinal logistic regression models were performed (models 2 and 3). For the first adjusted model, model 2, confounders included cleft type (cleft lip only / cleft palate with or without cleft lip), syndromic status inclusive of children with non-syndromic PRS, biological sex, SES, hearing status, and child's age at completion of the 18-month questionnaire. The second adjusted model, model 3, was restricted to children who were born with CP+/-L and was adjusted for syndromic status inclusive of children with non-syndromic PRS, biological sex, SES, hearing status child's age at completion of the 18-month questionnaire, and child's age at the time of primary palatal surgery.

### Secondary Analyses

Descriptive analysis was undertaken on SLT judgements of receptive language, expressive language, active cleft speech characteristics, number of different consonants heard at assessment, location of assessment and ability to rate velopharyngeal function for speech (ie, whether a judgement was made regarding presence or absence of resonance and/or airflow concerns—there was an option on the data collection form for SLTs to select ‘unable to rate’ for these features), and/or presence of passive cleft speech characteristics (eg, weak/nasalized consonants, nasal realization of plosives). Fisher's exact tests were used to determine if there was an association between location of assessment and pandemic grouping, and between SLT judgement of expressive language development and pandemic grouping. Unadjusted and adjusted binary logistic regressions were also performed to determine if there was an association between expressive language rating and pandemic grouping. Both unadjusted and adjusted Poisson regression was undertaken to determine whether there were differences in the number of consonants reported between the pre-pandemic and pandemic impacted groups. Binary logistic regressions, both unadjusted and adjusted, were undertaken to determine whether the ability to rate velopharyngeal function was associated with the location of the assessment. Adjusted models for the outcomes expressive language and number of consonants were adjusted for child's biological sex, child's age at assessment, and child's age at palate repair. The adjusted model for the outcome ability to rate velopharyngeal function was adjusted for child's age at assessment.

All analyses were performed using STATA/MP v18.0.^34^

## Results

### Participant Sample Characteristics

The total sample for the primary analysis comprised 778 children from the Cleft Collective Birth Cohort, of which 85.5% (n = 665) had reached 18 months of age prior to the pandemic. These children were all participants in the main Cleft Collective Birth Cohort study. Children born with CP+/-L accounted for 77.4% of the total sample (n = 602). Distribution of cleft subtypes was similar in both groups with, pre-pandemic group: cleft lip only n = 151 (23.1%), cleft palate only n = 248 (37.9%), unilateral cleft lip and palate n = 181 (27.6%), bilateral cleft lip and palate n = 75 (11.5%), and pandemic impacted group: cleft lip only n = 25 (22.7%), cleft palate only n = 43 (39.1%), unilateral cleft lip and palate n = 31 (28.2%), bilateral cleft lip and palate n = 11 (10.0%). Three participants had been specified as having a cleft involving the palate, but lip involvement was not specified. Due to small numbers in the pandemic impacted groups, cleft subtypes involving the palate were combined into a CP+/-L group. Children who had a syndrome (not including non-syndromic PRS) accounted for 12.3% of the total sample (n = 96). When children with non-syndromic PRS were combined with the syndromic category, 22.2% of the sample (n = 173) had a syndrome or non-syndromic PRS. Data on biological sex were available for 774 children (99.5%) with males representing 58.3% (n = 451) of the reduced sample. The median age of a child at the point of response to the 18-month parent questionnaire was 19.0 months (interquartile range: 18.6-20.2 months). All sample characteristic distributions for the pre-pandemic and pandemic impacted groups, including SES, hearing loss and age at primary palate repair, can be seen in Table 1.

**Table 1.** Sample Characteristics and Comparison Between Children in the Pre-Pandemic Group and Children in the Pandemic Impacted Group.

		Affected by the pandemic?	
	Overall n (%)	No, 18 months pre-pandemic n (column %)	Yes, affected by pandemic pre-18 months n (column %)
**Affected by pandemic?**	**778**	665 (85.5%)	113 (14.5%)
**Cleft type (n = 778)**			
Cleft lip only	176 (22.6%)	151 (22.7%)	25 (22.1%)
Cleft palate ± lip	602 (77.4%)	514 (77.3%)	88 (77.9%)
*χ^2^ test*		*χ^2 ^= 0.019; P = 0.891*	
**Syndromic status**	**778**		
Non-syndromic	682 (87.7%)	577 (86.8%)	105 (92.9%)
Syndromic (excluding PRS)	96 (12.3%)	88 (13.3%)	8 (7.1%)
*χ^2^ test*		*χ^2 ^= 3.381; P = 0.066* ^a^	
**Syndrome or PRS present**	**778**		
None present	605 (77.8%)	512 (77.0%)	93 (82.3%)
Presence of syndrome or PRS	173 (22.2%)	153 (23.0%)	20 (17.7%)
*χ^2^ test*		*χ^2 ^= 1.574; P = 0.210* ^a^	
**Biological sex**	**774**		
Male	451 (58.3%)	384 (57.9%)	67 (60.4%)
Female	323 (41.7%)	279 (42.1%)	44 (39.6%)
*χ^2^ test*		*χ^2 ^= 0.233; P = 0.629*	
**Index of multiple deprivation tertiles**	**676**		
1-3 (most deprived)	155 (22.9%)	137 (23.0%)	18 (22.8%)
4-7	251 (37.1%)	226 (37.9%)	25 (31.7%)
8-10 (least deprived)	270 (39.9%)	234 (39.2%)	36 (45.6%)
*Ordinal χ^2^ test*		*Ordinal χ^2 ^= 0.496; P = 0.974*	
**Hearing loss or impairment**	**735**		
No	611 (83.1%)	538 (84.2%)	73 (76.0%)
Yes	124 (16.9%)	101 (15.8%)	23 (24.0%)
*χ^2^ test*		*χ^2 ^= 3.955; P = 0.047* ^a^	
	**Median (IQR)**	**Median (IQR)**	
**Child's age at completion of ASQ-3 (age in months) (n = 730)**	19.0 (18.6–20.2)	19.1 (18.6–20.2)	18.8 (18.4–20.2)
*Mann-Whitney U*		*z = 1.798; P = 0.072*	
**Child's age at palate repair (age in months) (n = 583)**	9.5 (7.0–11.6)	9.2 (6.7–11.2)	11 (9.0–13.0)
*Mann-Whitney U*		*z = -4.110; P < 0.001* ^b^	

^a^ Differences in the proportion of children with a syndrome or with hearing loss between the pre-pandemic group and the pandemic impacted group are likely to reflect later diagnoses rather than rates of these conditions decreasing with time.

^b^ Difference in age at palate repair between pre-pandemic and pandemic impacted groups may reflect delays to primary surgery due to the pandemic.

Abbreviations: PRS, Pierre Robin Sequence; IQR, interquartile range; *χ^2^,* Chi-Squared; ASQ-3, Ages and Stages Questionnaire third edition; P, P-value.

The total sample for the secondary analysis, which used data from SLT assessments at ages 18-24 months, comprised 201 children; 183 (91.0%) in the pre-pandemic group and 18 (9.0%) in the pandemic impacted group (Table 2). These children were all participants in the Cleft Collective Speech and Language study. All children within the sample used for the secondary analysis were born with CP+/-L and comprised slightly more males than females (n = 111, 55.2%).

**Table 2.** Sample Characteristics for Analysis of Speech and Language Assessment Data Compared Between Children Who Had Received Their 18- to 24-Month SLT Assessment Pre-Pandemic and Children Who Will Have Experienced Social Restrictions Due to The Pandemic Before Their 18- to 24-Month Assessment.

	Affected by the pandemic?	
	No n (%)	Yes n (%)
**Overall (n = 201)**	183 (91.0%)	18 (9.0%)
**Location of speech and language appointment (n = 201)**		
Clinic (regional or local), home or other	183 (100%)	8 (44.4%)
Virtual	0	10 (55.6%)
*Fisher's Exact test*	*P < 0.001*	
**Expressive language (n = 193)**		
Age appropriate	86 (52.8%)	10 (55.6%)
Cause for concern	77 (47.2%)	8 (44.4%)
Unable to rate	12	0
*Fisher's Exact test (excluding ‘Unable to rate’)*	*P = 1.000*	
**Velopharyngeal function (n = 180)**		
No evidence of velopharyngeal dysfunction	51 (31.5%)	<5^a^
Evidence of velopharyngeal dysfunction	59 (36.4%)	<5^a^
Unable to rate^a^	52 (32.1%)	12 (66.7%)
	**Median (interquartile range)**	
**Number of consonants heard within speech and language assessment (n = 186)**	5 (3-8)	5 (3-6)
**Confounders**		
**Biological sex (n = 201)**	**n (%)**	**n (%)**
Male	101 (55.2%)	10 (55.6%)
Female	82 (44.8%)	8 (44.4%)
	**Median (interquartile range)**	
**Age (in months) at speech and language assessment (n = 195)**	23.4 (19.2–24.9)	18.6 (18.3–19.6)
**Age (in months) at palate repair (n = 200)**	9.0 (6.5 - 11.0)	11.0 (9.0 - 14.1)

^a^ Only the “Unable to rate” category could be reported due to small cell counts. Categories not presented include: “No evidence of velopharyngeal dysfunction” and “Evidence of velopharyngeal dysfunction.”

SLT, speech and language therapy.

Participants in the secondary analysis were those who had SLT assessment data at ages 18-24 months. Some of these participants did not have data available for use in the primary analysis, and for this reason, the sample used in the secondary analysis is not simply a subsample of that used in the primary analysis.

### Primary Analysis: Parent Questionnaire ASQ-3 Communication Domain Categorization Between pre-Pandemic and Pandemic Impacted Groups

Overall, more than half of the sample, 55.9% (n = 435), were categorized as being “On schedule” within the communication domain of ASQ-3. The remainder of the sample were categorized as “requires monitoring” (31.4%, n = 244) or “requires onward referral” (12.7%, n = 99). The distributions of the ASQ-3 communication domain between the pre-pandemic and pandemic impacted children appeared to be similar (Table 3), and the unadjusted ordinal logistic regression model (model 1) suggested there was no statistical evidence for a difference in the distributions of the ASQ-3 communication domain between the pre-pandemic and pandemic impacted children (n = 778; OR_unadjusted_ 1.11; 95% CIs 0.753, 1.629; *P* = 0.603).

**Table 3.** Distributions of ASQ-3 Communication Domain Categorization Between Pre-Pandemic and Pandemic Impacted Children.

		ASQ-3 communication domain		
Affected by pandemic? (n = 778)	Overall n (%)	On schedule n (row %)	Requires monitoring n (row %)	Requires onward referral n (row %)
No, 18 months pre-pandemic	665 (85.5%)	374 (56.2%)	208 (31.3%)	83 (12.5%)
Yes, affected by pandemic pre-18 months	113 (14.5%)	61 (54.0%)	36 (31.9%)	16 (14.2%)
		*Ordinal χ^2 ^= 0.299; P = 0.861*		

Distribution of ASQ-3 communication domain categorization for variables included as confounders for the primary analysis differed between whether children had a cleft involving the palate or not, a syndrome/non-syndromic PRS or not, biological sex, SES, hearing status, child's age at completion of 18-month questionnaire and age at palate repair (see Table 4). Syndromic status (excluding non-syndromic PRS) was not included as a confounder within either of the adjusted models, however, is presented within Table 4 for descriptive purposes. The first adjusted ordinal logistic regression model, model 2, supported the unadjusted (model 1) result, with no evidence for a difference in the distributions of the ASQ-3 communication domain categorization between the pre-pandemic and pandemic impacted groups (n = 631; OR_adjusted_ 0.96; 95% CIs 0.533, 1.742; *P* = 0.902). When restricting the analysis to CP+/-L cases only and further adjusting for age at palatal repair, model 3, a similar conclusion was drawn with no statistical evidence to suggest a in the distributions of the ASQ-3 communication domain between the pre-pandemic and pandemic impacted groups (n = 470; OR_adjusted_ 0.89; 95% CIs 0.433, 1.812; *P* = 0.741). Full regression models are presented in supplementary material, Table S1.

**Table 4.** Distribution of ASQ-3 Communication Domain Categorization for Variables Included as Confounders for the Primary Analysis.

		ASQ-3 communication		
	Overall n (%)	On schedule n (row %)	Requires monitoring n (row %)	Requires onward referral n (row %)
**Cleft type**	**778**			
Cleft lip only	176 (22.6%)	129 (73.3%)	35 (19.9%)	12 (6.8%)
Cleft palate ± lip	602 (77.4%)	306 (50.8%)	209 (34.7%)	87 (14.5%)
*Ordinal χ^2^ test*		*Ordinal χ^2 ^= 24.649; P < 0.001*		
**Syndromic status** ^a^	**778**			
Non-syndromic	682 (87.7%)	395 (57.9%)	218 (32.0%)	69 (10.1%)
Syndromic (excl Robin sequence)	96 (12.3%)	40 (41.7%)	26 (27.1%)	30 (31.3%)
*Ordinal χ^2^ test*		*Ordinal χ^2 ^= 23.499; P < 0.001*		
**Syndrome or PRS present**	**778**			
None present	605 (77.8%)	358 (59.2%)	191 (31.6%)	56 (9.3%)
Presence of syndrome or PRS	173 (22.2%)	77 (44.5%)	53 (30.6%)	43 (24.9%)
*Ordinal χ^2^ test*		*Ordinal χ^2 ^= 24.619; P < 0.001*		
**Biological sex**	**774**			
Male	451 (58.3%)	225 (49.9%)	155 (34.4%)	71 (15.7%)
Female	323 (41.7%)	210 (65.0%)	86 (26.6%)	27 (8.4%)
*Ordinal χ^2^ test*		*Ordinal χ^2 ^= 19.084; P < 0.001*		
**Index of multiple deprivation tertiles**	**676**			
1-3 (most deprived)	155 (22.9%)	70 (45.2%)	53 (34.2%)	32 (20.7%)
4-7	251 (37.1%)	137 (54.6%)	85 (33.9%)	29 (11.6%)
8-10 (least deprived)	270 (39.9%)	171 (63.3%)	79 (29.3%)	20 (7.4%)
*Ordinal χ^2^ test*		*Ordinal χ^2 ^= 19.780; P < 0.001*		
**Hearing loss or impairment**	**735**			
No	611 (83.1%)	356 (58.3%)	184 (30.1%)	71 (11.6%)
Yes	124 (16.9%)	53 (42.7%)	48 (38.7%)	23 (18.6%)
*Ordinal χ^2^ test*		*Ordinal χ2 = 10.364; P = 0.006*		
	**Median (IQR)**	**Median (IQR)**		
**Child's age at completion (age in months) (n = 730)**	19.0 (18.6–20.2)	19.3 (18.6–20.3)	18.8 (18.5–19.9)	18.9 (18.5–19.8)
*Kruskal-Wallis tests*		*χ^2 ^= 15.410; P < 0.001*		
**Child's age at palate repair (age in months) (n = 583)**	9.5 (7.0–11.6)	9.0 (6.9–11.0)	9.6 (6.5–11.4)	11.2 (7.8–13.5)
*Kruskal-Wallis tests*		*χ^2 ^= 14.529; P < 0.001*		

^a^ Variable not included as a confounder within fully adjusted ordinal logistic regression model. Sample characteristics provided for descriptive purposes.

Abbreviations: PRS, Pierre Robin Sequence; IQR, interquartile range; *χ^2^*, Chi-Squared; ASQ-3, Ages and Stages Questionnaire third edition; P, P-value.

### Secondary Analyses: Location of SLT Assessments

Data on the location of the child's 18- to 24-month SLT assessment were available for all 201 children. All children in the pre-pandemic group were reported to have received their assessments at a clinic, at the family home or the appointment location had been classified as “other.” Some specified via free text that these “other” locations were in other in-person contexts; however, the “other” location was not specified via free text in all cases in the pre-pandemic group. For children who had been affected by the pandemic, 44.4% (n = 8) had their appointment location recorded as at a clinic or at the family home, whereas for 55.6% (n = 10), the location had been recorded as “other” but for all 10, it was specified via free text that the appointment had been conducted virtually. The data for the number of virtual appointments therefore relied on SLTs specifying via a free text box on the data collection form that this method was used as there was no specific option for virtual appointments on the form. It is possible therefore that the number of appointments conducted virtually could have been higher in the pre-pandemic group and were included in the “other” category without specifying via free text that it was conducted virtually. However, this was not a common method of conducting appointments in the UK prior to the pandemic; hence, it had not been included as an option on the data collection forms. The data collection forms have since been updated to include this option.

### Secondary Analyses: Receptive and Expressive Language Judgements

SLTs were able to rate expressive language for 100% (n = 18) of the pandemic impacted group and 93.1% (n = 163) of the pre-pandemic group (Table 2). Of the children whose expressive language was rated, just over half of the pre-pandemic and pandemic impacted children were rated as “age appropriate” for expressive language (52.8%, n = 86; 55.6%, n = 10, respectively). No association was found between expressive language and whether the child had or hadn’t been affected by the pandemic (pre-pandemic n = 163, pandemic impacted n = 18; Fisher's Exact = 1.00). Both the unadjusted (n = 181; OR_unadjusted_ 0.89; 95% CIs 0.336, 2.379; *P* = 0.822) and adjusted binary logistic regressions supported this finding (pre-pandemic n = 157, pandemic impacted n = 18; OR_adjusted_ 0.66; 95% CIs 0.224, 1.947; *P* = 0.452). Due to small numbers in the pandemic impacted group, it was not possible to report outcomes relating to receptive language.

### Secondary Analyses: Velopharyngeal Function and Cleft Speech Characteristics

Speech and language therapists were unable to make a judgement about the presence or absence of speech features of velopharyngeal dysfunction for 32.1% (n = 52) of children in the pre-pandemic group and 66.7% (n = 12) of children impacted by the pandemic (Table 2). Due to small numbers in the pandemic impacted group, further analysis of SLT judgements of velopharyngeal function and cleft speech characteristics was not undertaken. When comparing ability to rate velopharyngeal function by location of appointment, weak evidence was found to suggest an increased odds of not being able to rate velopharyngeal function for those who have a virtual appointment (OR_unadjusted_ 4.63; 95%CIs 1.153, 18.562; *P* = 0.031: OR_adjusted_ 3.54; 95%CIs 0.849, 14.755; *P* = 0.083). The adjusted model was adjusted for age at SLT assessment and suggests a 254% increase in the odds of not being able to rate velopharyngeal function for speech for those who had a virtual appointment.

### Secondary Analyses: Size of Consonant Inventory

Data on consonant inventory size (the number of different consonants heard within the SLT assessment) were available for 186 children. The median number of different consonants heard for both pre-pandemic and pandemic impacted groups was 5 (Interquartile range 3-8 within the pre-pandemic group and 3-6 within the pandemic group) (Table 2). No differences in consonant inventory size between the pre-pandemic and pandemic impacted groups were found when performing unadjusted (pre-pandemic n = 169; pandemic impacted n = 17) and adjusted (pre-pandemic n = 163; pandemic impacted n = 17) Poisson regression models (IRR_unadjusted_ 0.89; 95%CIs 0.714, 1.122; *P* = 0.335: IRR_adjusted_ 0.98; 95%CIs 0.775, 1.249; *P* = 0.896). The adjusted analysis was adjusted for age at assessment, age at palate repair, and biological sex.

Full regression tables for all secondary analyses are available within supplementary materials (Table S2).

## Discussion

This study sought to understand whether pandemic-related social restrictions had impacted on the early speech and language skills of children with CL/P at age 18-24 months. Data from measures of speech and language development of children from before the pandemic was examined in comparison to children whose first 18-24 months had been impacted by pandemic-related restrictions. Primary analysis using data from the ASQ-3,^17^ completed as part of parent questionnaires sent to families participating in The Cleft Collective birth cohort study when child participants were aged 18 months, revealed no statistical evidence for a difference between pre-pandemic and pandemic impacted groups in ASQ-3 communication domain. When these analyses were adjusted for confounders (biological sex, cleft palate (yes/no), SES, syndromic or non-syndromic PRS, age at questionnaire completion and age at palate repair), there remained no evidence for a difference between pre-pandemic and pandemic impacted groups in either model 2 or model 3. From secondary analyses, there was no statistical evidence for a difference between pre-pandemic and pandemic impacted groups in SLT judgements of expressive language development or consonant inventory size at age 18-24 months, including when adjusted for biological sex, age at assessment, and age at primary palate repair. This may provide some reassurance for stakeholders in the early SLC development of children born with CL/P. Due to sample sizes in the pandemic impacted group, it was not possible to report on SLT judgements of receptive language, velopharyngeal function, or presence of cleft speech characteristics between the two groups. It cannot therefore be assumed from this study that there would be no difference between groups in these domains.

The findings reflect some studies of early language and communication development in samples of children from the general population^12,20^ although the participant samples are not directly comparable. However, other studies examining general population samples of children of the same age using the same measure (ASQ-3) have found differences in early language and communication skills between children who were and were not impacted by the pandemic.^19^ As described in the introduction, variation in how different studies define inclusion criteria for their pandemic impacted groups, variation in the nature and extent of pandemic-related social restrictions different study populations may have experienced, together with variation in pre-pandemic comparison groups and differences in participant demographics and characteristics could all contribute to the variation in findings. With the existing risks to speech and language development in children born with CP+/-L specifically, families may have made considerable efforts to support their child's communication development despite not having access to services or aspects of their cleft care as expected. Families of children born with CP+/-L may also have continued to receive some support from their regional specialist cleft center due to the established routine early support and monitoring pathways with clinical nurse specialists and SLTs, even if only by telephone or online/virtual video appointments.^27,28^ This may have supported parents to continue to facilitate their child's early SLC development irrespective of pandemic-related challenges.

It was not unexpected that a higher proportion of SLT assessments in the pandemic impacted group were reported to have been completed virtually (via online video platform). Evidence for an association between assessments being undertaken virtually and SLTs being unable to make a judgement about velopharyngeal function for speech was weak when adjusted for age at SLT assessment. However, the indication that virtual assessment may have some association with SLTs being less likely to be able to make a judgement regarding the presence or absence of speech features relating to velopharyngeal function reflects some parent perceptions from the early pandemic period that it may not always be possible to hear all speech features via this method of SLT delivery.^33^ This method of assessment was completely new to most SLTs in the UK at the time of data collection^34^ so clinicians, families, and services may have developed skills, resources, equipment, and experience in this method of service delivery since the data collection for this study. However, this is an important aspect of SLT service provision for children born with CP+/-L and with the small sample sizes (resulting in reduced precision), and limitations of the data regarding location of assessments in this study, further investigation is indicated to understand when, how and for whom, virtual speech assessment is effective to inform future service provision for this population.

## Limitations

Small sample size in the pandemic impacted groups, particularly in the secondary analyses of SLT assessment data limit the extent of analyses that were feasible and appropriate and reduce the precision of the results so should also be considered in the interpretation of the findings. The measures used to provide data for speech and language development in this study also have their limitations. The ASQ-3 is a widely used, robustly developed screening questionnaire of child development including language/communication.^17^ However, when compared to more detailed language assessments, there is some evidence that it may under identify children with SLC needs.^35^ In addition, the SLT judgements relating to language may have been made via a range of methods including informal assessment, observation, and parent report. The analyses in this study therefore provide a comparison between groups assessed in broadly the same way to address the aims of this study but cannot be assumed to represent true prevalence of language difficulties in this population when detailed formal assessment of language is carried out.

There will be variability in duration and extent of exposure and impact of pandemic-related social restrictions within the pandemic impacted group. While this would be very difficult, if not impossible, to control for, it is important to acknowledge as a limitation to be considered when interpreting the findings. Further, in addition to the limitations stated earlier regarding the data collection for the number of virtual SLT assessments, data for some of the confounding variables in the pandemic impacted group may have been less reliable due to the impacts of the pandemic. For example, distribution of syndromic and hearing status differed between the pre-pandemic and pandemic impacted groups which is perhaps likely to reflect reduced rates of diagnosis during the pandemic due to restrictions on in-person healthcare appointments, resulting in later identification of syndromic diagnoses and conductive hearing loss, rather than true reduced rates of these diagnoses over time. It is possible that some families may not have had access to a suitable device that would have enabled a virtual speech and language assessment, and some families may have also had problems with internet connectivity or no access to the internet at all. Data on digital poverty within our participants was not available and therefore we have not been able to account for this within our analyses.

## Implications for Clinical Practice and Future Research

The evidence from this research can be used to inform service providers and decision makers. Clinically, it remains important for individual children and families to be considered according to their specific needs and experiences in relation to any impacts of the pandemic.

In the event of any future lockdowns or similar restriction on the delivery of clinical care, both the benefits and limitations of parent report and virtual appointments should be considered. In particular, the challenges in carrying out assessment of velopharyngeal function virtually should be acknowledged and new techniques for investigating this should be explored if remote appointments continue to be required.

Future research is needed to investigate the use of cleft speech characteristics and understanding of language in a larger sample of pandemic impacted children born with cleft. The long-term impact on speech and language development in this high-risk population should also be explored and could be carried out using data from the Cleft Collective as the children mature.

## Conclusion

This study did not find statistical evidence that early speech and language development in children born with CL/P was impacted by experiencing the period of pandemic-related social restrictions. The finding that a high proportion of SLTs were unable to judge features of velopharyngeal function for speech in the pandemic impacted group in comparison to the pre-pandemic group may be related to delivering these 18- to 24-month assessments virtually via online video platform but this requires further work to examine and understand this issue.

The speech and language development of children born with CP+/-L should continue to be closely monitored and supported irrespective of the pandemic to facilitate early identification of concerns and appropriate intervention to maximize long-term life outcomes. Any impacts of experiencing the pandemic can continue to be monitored alongside this. Further work could examine long-term outcomes for children who experienced pandemic-related social restrictions.

## Supplemental Material

sj-docx-1-cpc-10.1177_10556656251328843 - Supplemental material for Impact of Pandemic-Related Social Restrictions on Language and Speech Development in Children with Cleft Lip and/or Palate at 18-24 Months Supplemental material, sj-docx-1-cpc-10.1177_10556656251328843 for Impact of Pandemic-Related Social Restrictions on Language and Speech Development in Children with Cleft Lip and/or Palate at 18-24 Months by Amy Davies, Lucy Southby, Sharon Baker, Helen Extence, Neil Brierley and Yvonne Wren in The Cleft Palate Craniofacial Journal
