# Supplementary material for: Impact of Pandemic-Related Social Restrictions on Language and Speech Development in Children with Cleft Lip and/or Palate at 18-24 Months
Source: Cleft Palate Craniofac J. 2025 Mar 28;63(5):1249–60. doi: 10.1177/10556656251328843 (PMC13076966; doi:10.1177/10556656251328843)
Supplement: sj-docx-1-cpc-10.1177_10556656251328843 - Supplemental material for Impact of Pandemic-Related Social Restrictions on Language and Speech Development in Children with Cleft Lip and/or Palate at 18-24 Months [file sj-docx-1-cpc-10.1177_10556656251328843.docx]

**Supplementary Materials**

Table S1 – Full regression results for the primary analyses, models 1-3. Pages 2 - 3

Table S2 – Full regression results for the secondary analyses. Pages 4 - 5

**Table S1 – Full regression results for the primary analyses, models 1-3.**

|  | Odds Ratio | P value | 95% confidence intervals | |
| --- | --- | --- | --- | --- |
| Model 1 - unadjusted analysis (n=778) |  |  |  |  |
| *Exposure* |  |  |  |  |
| Pre-pandemic | Reference category | | | |
| Affected by pandemic | 1.11 | 0.603 | 0.753 | 1.629 |
| *Cut points* |  |  |  |  |
| Cut 1 | 0.25 |  | 0.100 | 0.405 |
| Cut 2 | 1.94 |  | 1.722 | 2.160 |
| Model 2 - adjusted analysis (n=631) |  |  |  |  |
| *Exposure* |  |  |  |  |
| Pre-pandemic | Reference category | | | |
| Affected by pandemic | 0.96 | 0.902 | 0.533 | 1.742 |
| *Biological sex* |  |  |  |  |
| Male | Reference category | | | |
| Female | 0.39 | <0.001 | 0.275 | 0.544 |
| *Cleft type* |  |  |  |  |
| Cleft lip only | Reference category | | | |
| Cleft palate +/- lip | 1.94 | 0.002 | 1.288 | 2.934 |
| *Index of Multiple deprivation category* |  |  |  |  |
| 1 - most deprived | Reference category | | | |
| 2 - middle tertile of deprivation | 0.71 | 0.097 | 0.468 | 1.064 |
| 3 - least deprived | 0.41 | <0.001 | 0.271 | 0.620 |
| *Presence of a syndrome or non-syndromic PRS* |  |  |  |  |
| No | Reference category | | | |
| Yes | 2.52 | <0.001 | 1.702 | 3.739 |
| *Child's age at assessment* | 0.82 | 0.001 | 0.738 | 0.922 |
| *Cut points* |  |  |  |  |
| Cut 1 | -3.68 |  | -5.916 | -1.435 |
| Cut 2 | -1.75 |  | -3.981 | 0.480 |
| Model 3 - adjusted analysis (n=470) |  |  |  |  |
| *Exposure* |  |  |  |  |
| Pre-pandemic | Reference category | | | |
| Affected by pandemic | 0.89 | 0.741 | 0.433 | 1.812 |
| *Biological sex* |  |  |  |  |
| Male | Reference category | | | |
| Female | 0.36 | <0.001 | 0.246 | 0.537 |
| *Index of Multiple deprivation category* |  |  |  |  |
| 1 - most deprived | Reference category | | | |
| 2 - middle tertile of deprivation | 0.68 | 0.101 | 0.425 | 1.078 |
| 3 - least deprived | 0.40 | <0.001 | 0.249 | 0.637 |
| *Presence of a syndrome or non-syndromic PRS* |  |  |  |  |
| No | Reference category | | | |
| Yes | 2.22 | <0.001 | 1.438 | 3.440 |
| *Child's age at palate repair* | 1.08 | 0.004 | 1.024 | 1.138 |
| *Child's age at assessment* | 0.79 | <0.001 | 0.691 | 0.897 |
| *Cut points* |  |  |  |  |
| Cut 1 | -4.60 |  | -7.170 | -2.039 |
| Cut 2 | -2.65 |  | -5.197 | -0.103 |
| *Footnote: Model 1 – unadjusted and inclusive of all cleft types; Model 2 – adjusted and inclusive of all cleft types; Model 3 – adjusted and restricted to children born with cleft palate with or without lip to enable adjustment for timing of palatal repair.* | | | | |

**Table S2 – Full regression results for the secondary analyses.**

| Expressive language | | | | |
| --- | --- | --- | --- | --- |
|  | **Odds Ratio** | **P value** | **95% confidence intervals** | |
| Model 1 - unadjusted analysis (n=181) | |  |  |  |
| Pre-pandemic | Reference category | | | |
| Affected by pandemic | 0.89 | 0.822 | 0.336 | 2.379 |
| Model 2 - adjusted analysis (n=175) | |  |  |  |
| Pre-pandemic | Reference category | | | |
| Affected by pandemic | 0.66 | 0.452 | 0.224 | 1.947 |
| Biological sex |  |  |  |  |
| Male | Reference category | | | |
| Female | 0.46 | 0.017 | 0.247 | 0.871 |
| Child's age at palate repair | 1.00 | 0.915 | 0.919 | 1.099 |
| Child's age at assessment | 0.91 | 0.033 | 0.828 | 0.992 |
| Unable to rate velopharyngeal function | | | | |
|  | **Odds Ratio** | **P value** | **95% confidence intervals** | |
| Model 1 - unadjusted analysis (n=180) | |  |  |  |
| Location of appointment |  |  |  |  |
| In person | Reference category | | | |
| Virtual | 4.63 | 0.031 | 1.153 | 18.562 |
| Model 2 - adjusted analysis (n=174) | |  |  |  |
| Location of appointment |  |  |  |  |
| In person | Reference category | | | |
| Virtual | 3.54 | 0.083 | 0.849 | 14.755 |
| Child's age at assessment | 0.91 | 0.042 | 0.824 | 0.997 |
| Number of different consonants heard within the SLT assessment | | | | |
|  | **Incidence-Rate Ratio** | **P value** | **95% confidence intervals** | |
| Model 1 - unadjusted analysis (n=186) | |  |  |  |
| Pre-pandemic | Reference category | | | |
| Affected by pandemic | 0.89 | 0.335 | 0.714 | 1.122 |
| Model 2 - adjusted analysis (n=180) | |  |  |  |
| Pre-pandemic | Reference category | | | |
| Affected by pandemic | 0.98 | 0.896 | 0.775 | 1.249 |
| Biological sex |  |  |  |  |
| Male | Reference category | | | |
| Female | 1.04 | 0.596 | 0.911 | 1.177 |
| Child's age at palate repair | 1.02 | 0.035 | 1.001 | 1.038 |
| Child's age at assessment | 1.05 | <0.001 | 1.028 | 1.065 |
| *Acronyms: PRS – Pierre Robin Sequence; SLT – Speech and Language Therapist* | | | | |
